# Cancer and its predictors in Chinese adults with newly diagnosed diabetes and impaired glucose tolerance (IGT): a 30-year follow-up of the Da Qing IGT and Diabetes Study

**DOI:** 10.1038/s41416-022-01758-x

**Published:** 2022-03-07

**Authors:** Siyao He, Jinping Wang, Xiaoxia Shen, Xin Qian, Yali An, Qiuhong Gong, Bo Zhang, Bo Chen, Lihong Zhang, Xiaoping Chen, Yanyan Chen, Guangwei Li

**Affiliations:** 1 Endocrinology Centre, Fuwai Hospital, Chinese Academy of Medical Sciences and Peking Union Medical College, Beijing, China; 2 Department of Cardiology, Da Qing First Hospital, Da Qing, China; 3 Department of Endocrinology, China-Japan Friendship Hospital, Beijing, China; 4 Division of Non-Communicable Disease Control and Community Health, Chinese Center for Disease Control and Prevention, Beijing, China

**Keywords:** Risk factors, Type 2 diabetes, Cancer epidemiology

## Abstract

**Background:**

We aimed to explore if hyperglycaemia and hyperinsulinemia in the diabetes and prediabetes population were associated with increased risk of cancer occurence.

**Methods:**

Overall, 1700 participants with different glycaemic statuses were screened from the 110,660 residents of Da-Qing, China, in 1985. They were followed up to 30 years to access cancer outcomes.

**Results:**

Cancer was identified in 15.2% (259/1700) of the participants. The incidence of cancer in the normal glucose tolerance (NGT), impaired glucose tolerance (IGT) and diabetes groups was 6.06, 6.77, and 7.18 per 1000 person-years, respectively (*P* = 0.02). In the Fine-Gray model with all cause death as competing risk, compared with the NGT controls, both IGT and diabetes groups demonstrated significantly higher risk of cancer (for the IGT group, adjusted hazard ratio (aHR) = 1.77, 95% CI 1.38–2.27, *P* < 0.0001; for the diabetes, aHR = 3.34, 95% CI 2.64–4.22, *P* < 0.0001). Among the IGT participants, progress to diabetes (aHR = 2.28, 95%CI 1.24–4.20, *P* = 0.008) and insulin-area under the curve at baseline (for 1 SD increase, aHR = 1.39, *P* = 0.02) were also associated with the risk of cancer after adjustment of covariables.

**Conclusions:**

Hyperglycaemia in patients with diabetes, hyperinsulinemia, and progression to diabetes in people with IGT is significantly associated with the long-term increased risk of cancer occurrence.

## Background

Cancer is one of the primary non-communicable diseases and an important focus of public health. In 2021, Cancer Statistics reported that 1,898,160 new cancer cases and 608,570 cancer deaths were projected to occur in the United States [1]. In China, it is predicted that there will be about 4,292,000 newly diagnosed invasive cancer cases in 2015, corresponding to almost 12,000 new cancer diagnoses on average each day [2]. The GLOBOCAN reported that in China, in 2018, about 4.28 million new cancer cases were diagnosed, and 2.86 million cancer deaths occurred, which accounted for nearly 24% of all cancer cases and 30% of cancer deaths worldwide at the time [3].

As early as the 1960s, some population-based studies had found that populations with a history of type 2 diabetes had a higher risk of cancer [4]. In 2009, Vigneri et al. reported that people with diabetes showed an increased risk for liver, pancreas, endometrium, colon/rectum, and breast cancers [5]. There appears to be no association of diabetes with lung cancer. This lack of association with other cancers is inconclusive [6]. As for the underlying mechanism, hyperglycaemia, hyperinsulinemia, insulin resistance, and obesity are suspected to play a role. Recent studies on the relationships between hyperglycaemia, hyperinsulinemia/insulin resistance and cancer not only focused on patients with diabetes [5, 7–9], but also extended to participants with non-diabetic fasting hyperglycaemia and high glycated haemoglobin (HbA_1c_) [10]. However, results of these studies only involved indices related to basal glucose and insulin. The insulin area under the curve (insulin-AUC) during an oral glucose tolerance test (OGTT) is rarely studied due to the complexity of requiring multiple blood samples.

To the best of our knowledge, whether and how insulin resistance and blood insulin and glucose contribute to cancer development during the progression to diabetes from non-diabetic mild hyperglycaemia status has not been fully investigated. In particular, no studies on this topic with an inception cohort have been conducted in China. It is uncertain if the carcinogenic effect in the diabetes population is caused by insulin resistance, hyperinsulinemia, or hyperglycaemia itself. The purpose of the present study was to explore the predictors of cancer in Chinese adults who were diagnosed with diabetes or prediabetes, or had a normal glucose tolerance (NGT) in 1986, and to perform-ups up to 30 years.

## Methods

### Study design and participants

The 30-year follow-up of the Da Qing IGT and Diabetes Study was an observational cohort study that aimed to investigate the long-term outcomes, such as diabetes-related macro- and micro-vascular complications and cancer, and their related risk factors in a population with diabetes and impaired glucose tolerance (IGT). Details of the design, methods, and participants of the Da Qing IGT and Diabetes Study, as well as the major outcomes of the 30-year follow-up, have been reported elsewhere [11, 12]. Briefly, in 1985, 110,660 adults, accounting for approximately half of the residents who were aged 25 years and over in Da Qing, China, underwent screening for diabetes based on their 2-h plasma glucose level (PG2h), after a standardised breakfast. A total of 106,704 participants with a PG2h < 6.7 mmol/L were excluded from the study at the initial screening. The remaining 3956 participants with a PG2h ≥ 6.7 mmol/L underwent a 75-g OGTT to identify their glucose tolerance status. Based on the 1985 WHO criteria for the diagnosis of type 2 diabetes and IGT, 630 people were identified as newly diagnosed type 2 diabetes (NDM) and 576 people as IGT. Additional 520 participants with NGT who were age- and sex-matched with the IGT group were included as controls. All the participants of the present study had no history of cancer at enrolment. However, 19 people with NDM, one with IGT, and six with NGT were excluded due to missing information at any time point during OGTT at baseline, which left a total of 1700 participants (611 with NDM, 575 with IGT, and 514 with NGT) in the present analysis. The people with IGT participated in a 6-year randomised clinical trial of lifestyle intervention [11]. Once diabetes occurred, the patients started anti-diabetic treatment followed the guidelines of China Diabetes Society. A 75-g OGTT was repeated every 2 years to determine the glycaemic status of participants during the 6-year intervention period. At the end of the 6-year trial, all participants were informed of the results and asked to receive medical care from their usual medical providers.

### Data collection

At baseline (in 1986), data on demographic characteristics and lifestyle factors were collected from the participants by standardised interview. Clinical examination was performed to obtain blood pressure, height, weight, and other key indicators. Glycaemic related parameters, including plasma glucose and insulin levels at fasting state and 1-h and 2-h during OGTT were measured by trained staff and physicians at entry the study. The participants were then followed up for 30 years to determine if they had developed cancer. Cancer outcomes were collected in all the original study participants in the follow-up interviews, which were conducted at 20, 23, and 30 years after 1986. For deceased participants, we asked proxy informants about the date and hospital where the cancer was diagnosed. For all participants the medical records together with the informant interviews, were reviewed and adjudicated independently by two doctors to establish the underlying cause of cancer. Disagreements were resolved by a third senior physician. The diagnosis of cancer and site-specific cancer was based on the international classification of diseases (ICD) codes. Among the 259 cases of cancer, 187 were confirmed by the medical records, 21 cases were diagnosed by cancer hospitals in Beijing, Shanghai and other big cities in China, 45 cases were diagnosed by Daqing tertiary A hospital, where only 6 cases were physician diagnosed proxy self-reported. 25 people did not continue follow-up. To clarify if progression to diabetes and the medications for the treatment of hyperglycaemia associate with cancer risk, progression to diabetes was evaluated in people with NGT and IGT (either in the intervention or the non-intervention subgroups). Information regarding glucose-lowering drugs was also collected. 332 patients took oral hypoglycaemic agent(s) (OHA) alone, 234 patients took insulin alone and 444 patients took insulin plus OHA. Altogether, there were 1010 people who had accepted glucose lowing medication therapy.

Written informed consent was provided by either the study participants or the proxy who served as informants for deceased ones. Institutional review boards at the Chinese Centre for Disease Control and Prevention and Fuwai Hospital approved the trial and follow-up studies.

### Statistical analysis

Descriptive statistics were used to characterise the demographics, laboratory measures, and other key indicators of the study population. Continuous variables were expressed as the mean and standard deviation or median and interquartile range (IQR) depending on the distribution, and categorical variables as the frequency and percentage. The insulin area under the curve (AUC) during OGTT was calculated through trapezoidal estimation of the fasting and post-load plasma insulin levels with the following formula: insulin area = fasting insulin (mU/L)/2 + insulin 1 h (mU/L) + insulin 2 h (mU/L)/2. The homoeostatic model assessment for insulin resistance (HOMA_IR) was calculated using the following formula: HOMA_IR = [fasting glucose (mmol/l) × fasting insulin (mU/l)/22.5] [13]. Matsuda insulin sensitivity index was defined as the 1/square of the product of fasting insulin and fasting glucose times the product of mean insulin and mean glucose during OGTT [14]. We used the reverse of Matsuda insulin sensitivity index as the Matsuda insulin resistant index.

Analysis of variance was used to compare differences across groups for continuous variables and the chi-squared test or Fisher’s exact probability method (when the expected frequency was greater than 25% or less than 5%) for categorical variables. Incidence of cancer was calculated as the number of events divided by person-years of exposure censored at the time of diagnosis of cancer, loss to follow-up, death, or on 31 December 2016, whichever occurred first. The Kaplan–Meier method was used to determine the time-to-event survival curves for cancer for NDM, IGT, and NGT separately, and log-rank tests were used to compare the differences between NDM and NGT and between IGT and NGT. Standard Cox regression adjusting for age, sex, smoking status (smoker or non-smoker), systolic blood pressure (SBP), and body mass index (BMI) were first performed to assess the association of plasma insulin and glucose-related factors individually with cancer occurrence. Adjusted hazard ratios (aHR) and 95% confidence intervals (CI) were calculated. Fine-Gray model with all cause death as competing risk was used to explore if the participant with IGT or diabetes have a higher risk of cancer. Sub-group analyses were then performed to evaluate the association of insulin resistance, insulin level and progression to diabetes with cancer occurrence in IGT population. The significance level for all tests was set to 0.05 (two-sided). All data were prospectively analysed using SAS software, version 9.4 (SAS Institute Inc., Cary, NC, USA).

## Results

### Baseline characteristics of the study population

In addition to plasma glucose levels, almost all baseline parameters among participants in the three groups (NDM, IGT, and NGT), such as age, sex, BMI, blood pressure (SBP), and smoking status were significantly different. The fasting and post glucose load plasma insulin response were also different between the IGT and NGT groups (Table 1). Over a 30 years follow-up period (1986–2016), the cumulative incidence of diabetes was 76.9% in IGT-intervention group, 30.4% in the NGT group and 91.3% for the IGT non-intervention group.

**Table 1:** Characteristics of participants with different glycemic status.

	NGT (*n* = 514)	IGT (*n* = 575)	NDM (*n* = 611)	*P* value
*At baseline*				
Age (years)	44.0 ± 8.9	45.2 ± 9.3	48.3 ± 8.8	<0.0001
Sex (Male, %)	54.3	54.2	47.6	0.03
Smoker (%)	44.5	41.3	34.4	0.002
Obesity (%)	31.5	60.6	54.4	<0.0001
Fasting glucose (mmol/L)	4.8 ± 0.7	5.6 ± 0.8	8.6 ± 3.1	<0.0001
Glucose at 1 h (mmol/L)	6.7 ± 1.4	11.3 ± 2.0	16.0 ± 3.5	<0.0001
Glucose at 2 h (mmol/L)	5.0 ± 1.2	9.0 ± 0.9	15.3 ± 3.6	<0.0001
SBP (mmHg)	122 ± 21	133 ± 24	136 ± 24	<0.0001
DBP (mmHg)	82 ± 14	88 ± 14	88 ± 14	<0.0001
BMI (kg/M^2^)	23.7 ± 3.5	25.8 ± 3.8	25.5 ± 3.6	<0.0001
Fasting insulin (mu/L)	18.1 ± 14.5	24.5 ± 16.1	–	<0.0001
Insulin at 2 h (mu/L)	97.4 ± 57.5	123.9 ± 72.7	–	<0.0001
Insulin at 2 h-Fasting insulin (mu/L)	63.7 ± 51.2	125.5 ± 75.5	–	<0.0001
Insulin AUC (mu/L)^a^	136.8 ± 71.9	198.1 ± 103.3	–	<0.0001
*Over 30 years of follow-up*				
Cancer(*n*)	77	93	89	
Incidence of cancer per1000 person-years (95% CI)	6.06	6.77	7.18	0.02
Cancer(*n*)	(4.9–7.5)	(5.6–8.2)	(5.8–8.9)	
Cancer-free time (years)	26.2 ± 8.1	23.7 ± 9.1	19.2 ± 6.9	<0.0001

Data in the table are mean ± SD, unless otherwise indicated.

*BMI* body mass index, *CI* confidence interval, *DBP* diastolic blood pressure, *IGT* impaired glucose tolerance, *NDM* newly diagnosed diabetes, *NGT* normal glucose tolerance, *SBP* systolic blood pressure, *Cancer-free time* years from entry to the diagnosis of cancer.

^a^Insulin AUC, insulin area under the curve of 75 g glucose tolerance test was carried out in 507 participants including 200 cases in the NGT group and 307 cases in the IGT group.

### Association of diabetes status and plasma glucose levels with incidence of cancer in the whole study population and non-diabetes group alone

During a median follow-up of 27 years (IQR: 15–30 years), a total of 259 (15.2%) cancer cases were identified in all three groups. These included 131 cases (50.6%) of gastrointestinal tract and hepatobiliary cancers (including 74 cases of gastrointestinal tract cancer and 57 cases of liver, gallbladder, and pancreatic cancer), 66 cases (25.5%) of lung cancer, 35 cases (13.5%) of urogenital system cancer (including 20 gynaecological tumours), and 27 cases (10.4%) of other types of cancer, including bone, skin, brain, oral cavity, and haematological cancers. The incidence of cancer in the NGT, IGT, and NDM groups was 6.06, 6.77, and 7.18 per 1000 person-years, respectively(*P* = 0.02). (Table 1).

In the whole study participants, results of the Cox model analysis showed, after the adjustment of age, sex, BMI, SBP and smoking status, the plasma glucose levels at 1 and 2 h during the 75 g glucose load OGTT were significantly associated with an increased incidence of cancer (aHR = 1.03, 95% CI 1.01–1.06, *P* = 0.02 for 1 hour glucose, and aHR=1.03, 95% CI 1.004–1.06, *P* = 0.02 for 2 hours glucose), whereas the fasting plasma glucose was not (aHR = 1.01, 95% CI 0.96–1.07, *P* = 0.69). For the fluctuation of plasma glucose levels, one mmol increase in plasma glucose level at 1-h and 2-h during OGTT was also found to be correlated with a higher cancer risk (*P* < 0.003), whereas in the non-diabetes participants including NGT and IGT only an increment of 2 h glucose level was associated with the risk of cancer (*P* = 0.0049), but the diabetes-free time over 30 years of follow-up was inversely associated with the risk of cancer after the same adjustment (for 10 years increase, aHR = 0.75, 95% CI 0.64–0.87, *P* = 0.0002). As for the IGT participants alone, neither the fasting nor the increments of post glucose levels were associated with the cancer risk (Table 2).

**Table 2:** Association of plasma glucose related factors and insulin with cancer occurrence over 30-year.^a^

	Hazard ratio	95% Confidence interval	*P* value
*In all participants(n* *=* *1697)*			
Fasting glucose (mmol/L)	1.01	0.96–1.07	0.69
Glucose at 1 h (mmol/L)	1.03	1.01–1.06	0.02
Glucose at 2 h (mmol/L)	1.03	1.004–1.06	0.02
Glucose at 1 h-Fasting glucose(mmol/L)	1.06	1.02–1.11	0.003
Glucose at 2 h-Fasting glucose (mmol/L)	1.06	1.02–1.10	0.002
*In non-diabetes participants*^b^*(n* *=* *1085)*			
Fasting glucose (mmol/L)	0.91	0.77–1.15	0.54
Glucose at 1 h (mmol/L)	1.03	0.99–1.11	0.25
Glucose at 2 h (mmol/L)	1.05	0.99–1.14	0.17
Glucose at 1 h-Fasting glucose (mmol/L)	1.05	1.001–1.13	0.11
Glucose at 2 h-Fasting glucose (mmol/L)	1.09	1.01–1.19	0.0049
Diabetes-free time (10 years)	0.75	0.64–0.87	0.0002
*In participants with IGT(n* *=* *575)*			
Fasting glucose (mmol/L)	0.89	0.67–1.17	0.40
Glucose at 1 h (mmol/L)	1.01	0.91–1.11	0.90
Glucose at 2 h (mmol/L)	1.07	0.85–1.34	0.59
Glucose at 1 h-Fasting glucose (mmol/L)	1.03	0.92–1.15	0.62
Glucose at 2 h-Fasting glucose (mmol/L)	1.13	0.92–1.39	0.26
Progression to diabetes (yes = 1)^c^	2.28	1.24–4.20	0.008
Fasting insulin (mu/L) (*n* = 302)^d^	1.47	0.87–2.49	0.15
Insulin AUC (mu/L) (*n* = 302)^d^	2.03	1.08–3.81	0.03

*Insulin AUC* insulin area under the curve, *IGT* impaired glucose tolerance, *NDM* newly diagnosed diabetes, *NGT* normal glucose tolerance, *Diabetes-free time* number of years from entry to time of onset of diabetes.

^a^Age, sex, smoking status, systolic blood pressure and body mass index adjusted.

^b^Non-diabetes participants included people with IGT and NGT.

^c^Progression to diabetes was taken as a time-dependent variable in COX model.

^d^Log transformed value.

**Fig. 1:**
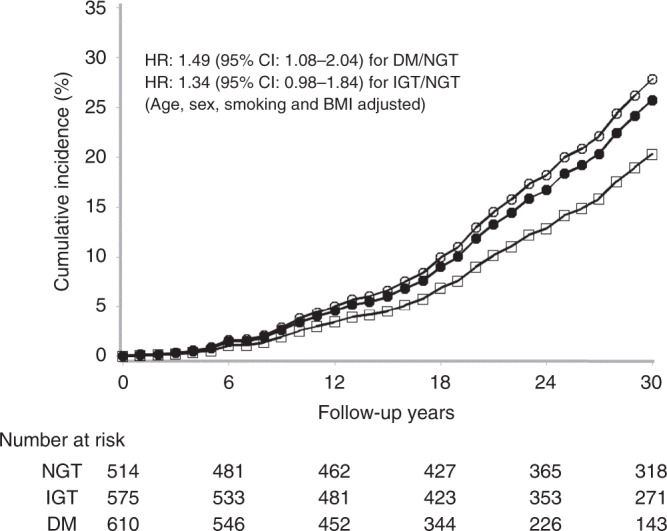
Cumulative incidence of cancer in NDM, IGT, and NGT groups over 30 years. Cumulative incidence of cancer. HRs were adjusted for age, sex, smoking status, and BMI. The white circles represent the diabetes group, the black circles represent the IGT group, and the squares represent the NGT group. HR, hazard ratio; IGT, impaired glucose tolerance; NDM: newly diagnosed type 2 diabetes; NGT, normal glucose intolerance.

Multivariable Cox regression analyses in the whole population also showed that baseline age (aHR = 1.07, 95% CI 1.05–1.08, *P* < 0.0001), male sex (aHR = 1.36, 95% CI 1.02–1.81, *P* = 0.04) and smoking status (aHR = 1.44, 95% CI 1.10–1.90, *P* = 0.009) were significantly associated with increased cancer incidence. After the adjustment of age, sex, BMI, and smoking status, the risk of cancer in the diabetes group was significantly associated with cancer occurrence (aHR = 1.49, 95% CI 1.08–2.04, *P* = 0.015) (Fig. 1). However, after further adjusting for SBP, the risk of cancer in diabetes group was only marginally higher than that in the NGT group (aHR = 1.36, 95% CI 0.99–1.88, *P* = 0.06) (Table 3) although the diabetes group showed 7.0 years of less cancer-free time than the NGT group (19.2 years vs. 26.2 years, *P* < 0.0001) (Table 1).

**Table 3:** Association of hyperglycemic status risk of cancer occurrence over 30 years in all participants.^a^

	HR	95% CI	*P* value
*Model 1.* Standard Cox			
Age (years)	1.07	1.05–1.08	<0.0001
Sex (male = 1)	1.36	1.02–1.81	0.04
BMI (kg/M^2^)	1.003	0.97–1.04	0.89
Smoking status (Yes = 1)	1.44	1.10–1.90	0.009
SBP (mmHg)	1.004	0.998–1.01	0.17
IGT vs NGT	1.22	0.89–1.68	0.21
DM vs NGT	1.36	0.99–1.88	0.06
*Model 2.* Fine-Gray models^a^			
Age (years)	1.07	1.06–1.08	<0.0001
Sex (male = 1)	1.20	0.99–1.45	0.06
BMI (kg/M^2^)	0.99	0.97–1.02	0.47
Smoking status (Yes = 1)	1.18	0.98–1.42	0.08
SBP (mmHg)	1.01	1.005–1.01	<0.0001
IGT vs NGT	1.77	1.38–2.27	<0.0001
DM vs NGT	3.34	2.64–4.22	<0.0001

*CI* confidence interval, *HR* hazard ratio, *IGT* impaired glucose tolerance, *DM* newly diagnosed diabetes, *NGT* normal glucose tolerance.

^a^All cause death as competing risk.

In the research on cancer risk, the competitive risk of death is a problem that needs to be considered. Some results of this study attracted our attention to this issue. It was observed that over the 30 years follow-up, all cause deaths and cardiovascular deaths increased more than twice across the NGT, IGT, and NDM groups (29.6% to 45.3% to 68.3% for all deaths and 11.9% to 22.4% to 34.2% for CVD death, respectively), while the number of cancer occurred in the people died across the three groups was gradually reduced (36.8% to 27.2% to 18.0%), (Supplementary Fig. 1). In addition, the median age of cancer occurrence in the NGT group (68 years, IQR 57–73 years) overlapped with age at death in the IGT group (68 years, IQR 62–76 years), or even higher than that in the NDM group (67 years, IQR 59–71 years) (Supplementary Table 1). These results highly suggest that the increased premature death caused by hyperglycaemia in people with IGT and diabetes may have masked the real risk of cancer. As expected, in the Fine-Gray model with all cause death as competing risk, it was found that both the IGT group (aHR = 1.77, 95% CI 1.38–2.27, *P* < 0.0001) and the diabetes group (aHR = 3.34, 95% CI 2.64–4.22, *P* < 0.0001) had significantly higher risk of cancer compared with the NGT group (Table 3).

### Association of insulin resistance, plasma insulin level, and progression to diabetes with cancer risk in participants with IGT

In the IGT group alone, the log-transformed insulin AUC level during OGTT was significantly associated with the risk of cancer (aHR = 2.03, 95% CI 1.08–3.81, *P* = 0.03) (Table 2). Furthermore, after controlling for age, sex, smoking status, BMI, SBP, and lifestyle intervention, the multivariable Cox analysis showed that one SD increase in the plasma insulin AUC still significantly associated with the risk of cancer occurrence (aHR = 1.39, 95% CI 1.05–1.84, *P* = 0.02), as was the one SD increase in the Matsuda insulin resistance index (aHR = 1.41, 95% CI 1.06–1.87, *P* = 0.02) (Table 4). However, the HOMA_IR was not be associated with the risk of cancer. Of note, in the models for assessing the association between insulin AUC or insulin resistance indices and cancer risk, a steady significant association between the progression to diabetes and cancer risk existed simultaneously (HR ranged from 1.87 (*P* = 0.001) to 2.30 (*P* < 0.0001) (Table 4). This association was confirmed by the Cox model analysis performed in 575 participants with IGT when progression to diabetes was taken as a time-dependent variable (HR = 2.28, *P* = 0.008) (Table 2).

**Table 4:** Predictive effects of progression to diabetes and one-SD increase in insulin resistance and insulin level at baseline on risk of cancer in participants with IGT over 30 years.

	HR	95% CI	*P* value
*Model 1*			
Age (9.3years)	2.89	1.87–4.46	<0.0001
Sex (male = 1)	1.43	0.69–2.97	0.34
Smoking status (yes = 1)	1.50	0.75–3.00	0.25
BMI (3.8 kg/M^2^)	1.40	0.96–2.06	0.08
SBP (23 mmHg)	1.22	0.83–1.81	0.32
Intervention (yes = 1)	0.88	0.40–1.94	0.76
Progressed to diabetes (yes = 1)	2.30	1.54–3.45	<0.0001
Matsuda_IR^a^ (170 mu.mmol/l)	1.41	1.06–1.87	0.02
*Model 2*			
Age (9.3 years)	2.73	1.85–4.03	<0.0001
Sex (male = 1)	1.13	0.57–2.25	0.72
Smoking status (yes = 1)	1.87	0.96–3.66	0.07
BMI (3.8 kg/M^2^)	1.44	1.02–2.03	0.04
SBP (23 mmHg)	1.31	0.94–1.82	0.11
Intervention (yes = 1)	1.03	0.50–2.13	0.93
Progressed to diabetes (yes = 1)	1.87	1.28–2.72	0.001
Homa_IR^b^ (4.34 mu/l)	1.15	0.85–1.53	0.37
*Model 3*			
Age (9.3 years)	2.91	1.89–4.50	<0.0001
Sex (male = 1)	1.43	0.69–2.96	0.33
Smoking status (yes = 1)	1.56	0.78–3.10	0.21
BMI (3.8 kg/M^2^)	1.40	0.97–2.02	0.07
SBP (23 mmHg)	1.28	0.87–1.88	0.21
Intervention (yes = 1)	0.88	0.40–1.92	0.74
Progressed to diabetes (yes = 1)	2.18	1.47–3.26	0.0001
Insulin area** (103 mu/l)	1.39	1.05–1.84	0.02

*CI* confidence interval, *HR* hazard ratio, *IGT* impaired glucose tolerance, *SD* standard deviation.

^a^Matsud_IR was an insulin resistance index; Insulin AUC, the insulin area under curve in OGTT.

^b^Only participants with IGT having insulin determination were included (*n* = 307).

### Influence of glucose-lowering treatment on the risk of cancer

Use of insulin and OHA or either of the two alone was not associated with an increased risk of cancer occurrence (aHR = 1.04, 95% CI 0.65–1.68, *P* = 0.87), comparing to those who did not use these medications. Similarly, participants using insulin or insulin plus OHA were also not associated with a higher cancer risk (aHR = 0.88, 95% CI 0.50–1.57, *P* = 0.67) compared with those using OHA alone (Supplementary Table 2).

## Discussion

In this prospective population-based cohort of Chinese adults, participants with diabetes or prediabetes back in 1985 were associated with significantly increased risk of cancer over a follow-up of up to 30 years.

Literature review suggests that large cohort studies with multiple pre-diagnostic biospecimens, which allows the investigation of the mechanism between diabetes and cancer, such as whether hyperglycaemia itself in diabetes or other factors concomitant with high plasma glucose had induced a high cancer risk, are rare. In this population-based cohort study, a total of 1700 participants underwent an OGTT at baseline, and several hundreds of them had fasting and post-load insulin records were available. Moreover, the participants were followed up for up to 30 years, allowing enough time for the detection of new cancer cases. We, therefore, believe that this study provides unique data on the predictors of cancer and the association of diabetes onset, hyperglycaemia, and hyperinsulinemia with cancer development.

### Relationship between diabetes status, blood glucose, and the risk of cancer

The association of hyperglycaemia, such as higher HbA_1c_, with a higher incidence of all cancers remains unclear. One study even found a U-shaped relationship between cancer and HbA_1c_ level [15]. A large population sample found a linear effect of both fasting and post-challenge glucose on cancer mortality, and the result did not change even after the adjustment for fasting insulin [16]. Interestingly, in vivo models that demonstrated a reduced tumour growth in the setting of type 1 diabetes suggested that hyperglycaemia does not lead to increased neoplastic growth, at least in the setting of insulin deficiency [17]. It is still unclear whether high blood glucose levels reaching the overt diabetes standard is a prerequisite for a higher risk of cancer. In the present study, we were able to detect a gradual increase in the incidence of malignant tumours across the three groups with different glycaemic statuses. The standard Cox analysis found, compared with the NGT group, the IGT group had not shown an increased risk of cancer (*P* = 0.22) and the risk was only marginally increased in the diabetes group (*P* = 0.06) after adjusting for traditional confounders. This risk was greatly increased after competing all cause death in the Fine-Gray model, the cancer risk in IGT and diabetes groups was increased to 1.77-fold and 3.34-fold respectively (*P* < 0.0001) compared with NGT. Our data revealed that once patients with IGT progressed to diabetes, the risk of cancer was doubly increased. These findings suggested that hyperglycaemia, when reaching a certain level will induce a stronger carcinogenic effect.

Is there any difference between fasting glucose, post-load glucose levels and the risk of cancer? Our study revealed among all participants, the absolute post-load blood glucose levels and the increment of post-challenge glucose levels, but not fasting glucose levels, were significantly correlated with a higher risk of cancer. Our data also demonstrated that the increase of plasma glucose levels in a population with broad glucose spectrum, such as in all the participants with diabetes, IGT and NGT, showed a significant association with the occurrence of cancer, but this association is not seen in people with a narrow blood glucose spectrum such as in the IGT group alone or in the IGT plus NGT group. It suggested that the hyperglycaemia may have carcinogenic effects and controlling excessive blood glucose fluctuation after meals may be helpful for cancer prevention in diabetes patients.

### Relationship of insulin resistance and insulin levels with cancer risk among participants without diabetes

Whether insulin is related to various cancer types has attracted the attention of many researchers. A 9.6-year case-cohort study of Finnish men showed that higher fasting insulin level was associated with an increased lung cancer risk [18]. Another study in type 2 diabetes found that hyperinsulinemia can promote breast cancer cell proliferation, migration, and invasion [19]. A meta-analysis including 16 studies and 14,007 patients found that higher levels of insulin were significantly associated with increased risk of colorectal adenomas [20]. Surprisingly, a 29-year cohort study found that elevated basal insulin level was a negative predictor of cancer prognosis [21]. Although some studies have shown the role of hyperinsulinemia in different cancer types [19–21], the long-term risk of exposure to high levels of insulin, especially to high levels of post-challenge insulin for cancer development is relatively understudied. Individuals with IGT are appropriate candidates for investigating the relationship between hyperinsulinemia and cancer, as most of them are likely to present post-challenge hyperinsulinemia for many years before progressing to diabetes. In this study, with fasting and post glucose load insulin data collected, we were able to identify the different associations between these insulin levels and cancer risk. After adjusting for confounding factors, we found that the increment of post-load plasma insulin levels from the fasting state (the post-load insulin AUC in OGTT), but not fasting plasma insulin levels, significantly associated with the increased risk of cancer.

We also assessed the role of insulin resistance in cancer development. Interestingly, it was observed that the Matsuda insulin resistance index, which was calculated from post-challenge insulin and glucose levels, significantly predicted cancer, whereas the insulin resistance index HOMA_IR calculated from fasting insulin and glucose levels was not associated with the risk of cancer. This finding indicates that the HOMA_IR may have led to underestimating the carcinogenic effects of insulin resistance.

A report of the United Kingdom Prospective Diabetes Study commented that one cannot exclude the possibility that there exists a subset of tumours for which hyperglycaemia confers a growth advantage, and therefore, appropriate therapy for diabetes may limit tumour growth [21]. However, the aggregate data suggest that insulin receptor activation may be a more important factor than hyperglycaemia in determining tumour growth [22]. Interestingly, findings in the IGT people alone in the present study, the insulin AUC, but not plasma glucose levels, was significantly associated with the risk of cancer, suggesting in the prediabetes stage, the long-term exposure to hyperinsulinemia is likely an earlier and long-lasting important contributor to the development of cancer since in the IGT population hyperinsulinemia may exist for many years before the onset of diabetes. Even though, our data still showed it seems that progression to diabetes had a stronger influence on the cancer risk than insulin resistance/hyperinsulinemia. This highlights the potential benefit of preventing or delaying diabetes for the prevention of cancer in the high-risk individuals. The findings that both hyperinsulinemia and progression to diabetes from prediabetes status were significantly associated with the development of cancer, highlighted that only a perfect combination of preventing diabetes and eliminating hyperinsulinemia/correcting insulin resistance can prevent cancer effectively. Although the mechanism of cancer development in the general population is very comprehensive, approximately 40% of the risk factors of cancer are attributed to environmental and lifestyle conditions, which can be preventable in China and worldwide [3, 23, 24]. Results of the present study showed that the older aged patients that smoked, patients with diabetes or prediabetes, patients with coexisting hyperinsulinemia and insulin resistance, should be prioritised candidates for cancer prevention.

One study had reported that diabetes therapy was not associated with increased risk of the development of cancer due to the net effect of an increased risk caused by insulin and a decreased risk caused by the reduction of hyperglycaemia [24]. Data in the present study was consistent with this report. We observed that the use of insulin and oral glucose-lowering medications was not significantly associated with the risk of cancer.

In the interpreting of our study, several strengths and limitations deserve careful consideration. The strengths include long-term follow-up of participants from a large cohort. Participants were recruited from a population-based screening for diabetes in 110,660 Chinese adults, which accounted for half of the residents in the Daqing area, China in 1985, then were followed up for 30 years, which provided a long-term perspective on the occurrence of cancer and progression to diabetes. Second, the study analysed prospectively collected fasting and post-load glucose blood sample data as well as other data regarding potential confounders in participants with different glycaemic statuses. Third, we were able to analyse data from the IGT group and compare the results in cases of progression to diabetes and reversal to an NGT status. Therefore, we had the opportunity to evaluate the effects of prevention or delay of diabetes on the development of cancer.

A major limitation is the relatively small size of NDM and IGT individuals, which did not allow us to investigate the risk in relation to different subgroups of cancer. Another limitation is that in some participants, blood insulin levels were not measured due to a limited budget. Well-designed large-scale prospective epidemiological studies, such as the Diabetes Prevention Program Outcomes Study, are needed to confirm the association between diabetes, especially among participants with impaired glucose control, and the increased risk of cancer.

In conclusion, patients with diabetes and prediabetes had an increased risk of cancer. Among participants with IGT, insulin resistance, compensated hyperinsulinemia (indicating a high insulin demand) together with the progression to diabetes accelerated the development of cancer. It suggests, appropriate blood glucose-lowering treatment in patients with overt diabetes, and the prevention or delay of diabetes onset by eliminating high insulin demand among the prediabetes population, may benefit the reduction of cancer risk.

### Reporting summary

Further information on research design is available in the Nature Research Reporting Summary linked to this article.

## Supplementary information

SUPPLEMENTAL MATERIAL

Reporting Summary

## Data Availability

The data generated or analysed in this study will be provided upon reasonable request.
