# Supplementary material for: Cancer and its predictors in Chinese adults with newly diagnosed diabetes and impaired glucose tolerance (IGT): a 30-year follow-up of the Da Qing IGT and Diabetes Study
Source: Br J Cancer. 2022 Mar 7;127(1):102–8. doi: 10.1038/s41416-022-01758-x (PMC9276667; doi:10.1038/s41416-022-01758-x)
Supplement: Supplementary file 1 — SUPPLEMENTAL MATERIAL [file 41416_2022_1758_MOESM1_ESM.docx]

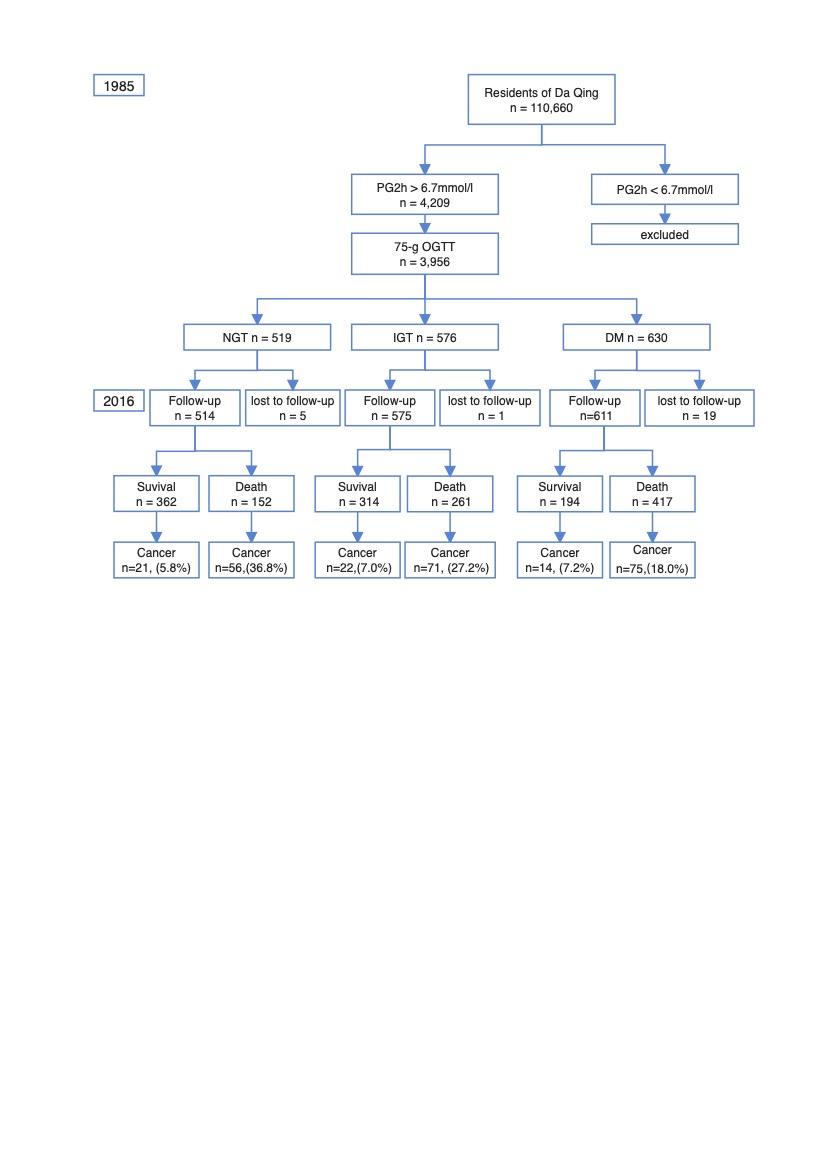
Supplementary Figure 1. Flowchart of the study

**Supplementary Table 1. Distribution of cancer in CVD death and all-cause death in Chinese adults with different glycemic status over 30 years**

|  | NGT  (n = 514) | IGT  (n = 575) | NDM  (n =611) | *P-*value* |
| --- | --- | --- | --- | --- |
| Any death, n (%) | 152 (29.6%) | 261 (45.3%) | 417 (68.3%) | < 0.0001 |
| CVD death, n (%) | 61 (11.9%) | 129 (22.4%) | 209 (34.2%) | < 0.0001 |
| Cancer/CVD death, n (%) | 21/61(33.4%) | 22/129(17.1%) | 14/209((6.7%) | < 0.0001 |
| Cancer/any death, n (%) | 56/152(36.8%) | 71/261 (27.2%) | 75/417 (18.0%) | < 0.0001 |
| Age at cancer diagnosis,  median (IQR) | 68 (57-73) | 68 (62-76) | 67 (59-71) | 0.19 |
| Age at non-cancer death,  median (IQR) | 70 (62-75) | 68 (61-74) | 67 (60-72) | 0.0003 |

CVD, cardiovascular disease; IGT, impaired glucose tolerance; IQR, interquartile range; NGT, normal glucose intolerance

**P*-value for comparison across three groups;

***P*-value: comparison between NGT and DM groups

**Supplementary Table 2. Association of anti-diabetes medication with risk of cancer occurrence in all participants**

|  | **HR** | **95% CI** | **P value** |
| --- | --- | --- | --- |
| **Model 1** |  |  |  |
| Age (year) | 1.05 | 1.02-1.08 | 0.0003 |
| Sex (male=1) | 1.80 | 1.13-2.86 | 0.01 |
| BMI (kg/m^2^) | 1.04 | 0.97-1.10 | 0.27 |
| SBP (mmHg) | 1.00 | 0.99-1.02 | 0.41 |
| Smoking status (yes=1) | 1.13 | 0.72-1.77 | 0.61 |
| Use of OHA and/or insulin (yes=1) | 1.04 | 0.65-1.68 | 0.87 |
| **Model 2** |  |  |  |
| Age (year) | 1.05 | 1.02-1.09 | 0.003 |
| Sex (male=1) | 1.36 | 0.80-2.32 | 0.26 |
| BMI (kg/m^2^) | 1.02 | 0.95-1.10 | 0.61 |
| SBP (mmHg) | 1.01 | 0.997-1.02 | 0.17 |
| Smoking status (yes=1) | 1.09 | 0.63-1.89 | 0.76 |
| Use of OHA vs OHA plus insulin (yes=1) | 0.88 | 0.50-1.57 | 0.67 |

BMI, body mass index; CI, confidence interval; HR, hazard ratio; OHA, oral hypoglycemic agents
